# Association between Educational Attainment and Risk of Postnatal Depression: Findings from the Czech Republic

**DOI:** 10.1192/j.eurpsy.2024.1677

**Published:** 2024-08-27

**Authors:** M. Kuklová, K. Hrdličková, A. Horáková, H. Němcová, A. Šebela

**Affiliations:** ^1^Centre of Perinatal Mental Health, National Institute of Mental Health, Klecany; ^2^Department of Epidemiology; ^3^Department of Demography and Geodemography, Charles University, Prague, Czech Republic

## Abstract

**Introduction:**

Postnatal depression is a common mental health condition that affects women across the globe. Lower education is frequently considered to be linked to an increased likelihood of postpartum depression. Nevertheless, inconsistent epidemiological evidence has been reported concerning the associations between education and postpartum depression risk. This study investigates the correlation between education level and postpartum depression in the Czech Republic.

**Objectives:**

The aim of this study was to examine whether there is an association between educational attainment and the risk of postnatal depression in women who have recently given birth in the Czech Republic.

**Methods:**

Women aged 18-45, who spoke Czech and had an email address, and had given birth in the hospital were eligible to participate in the study. The research was conducted in the maternity unit, where a medical professional presented the opportunity to take part. All participants were screened using the Edinburgh Postnatal Depression Scale (EPDS), with the cut-off score of ≥10 showing increased of postpartum depression.

Education data was collected via self-reported questionnaires. Binary logistic regression was employed to calculate the odds ratio (OR) with 95% confidence intervals (CI) to assess the relationship between educational attainment and postpartum depression risk, with sociodemographic and health-related characteristics being stepwise adjusted.

**Results:**

Our study consisted of 3,739 postpartum respondents (mean age of 31 years). The prevalence of increased postpartum depression (≥10 EPDS points) was 22.7%. Compared to individuals with higher education (reference category), those with basic education had a higher risk of postpartum depression (OR 1.67; 95% CI 1.26–2.23; p<0.001), even after adjusting for all covariates (OR 1.55; 95% CI 1.08–2.22; p=0.017). Basic education was found to have the strongest association with an increased risk of postpartum depression, even when adjusted for covariates. The association between education and postpartum depression was explained by the covariates.

**Conclusions:**

Having only basic education is a significant risk factor for postpartum depression. Interventions to reduce the burden of postpartum depression ought to focus on individuals with low levels of education.

**Disclosure of Interest:**

None Declared

